# A Rare Case of Aggressive Infective Endocarditis Due to Corynebacterium striatum

**DOI:** 10.7759/cureus.44903

**Published:** 2023-09-08

**Authors:** Serkan Dilmen, Sahhan Kilic, Akin Torun

**Affiliations:** 1 Cardiology, Menemen Public Hospital, Izmir, TUR; 2 Department of Cardiology, Sultan Abdulhamid Training and Research Hospital, Istanbul, TUR

**Keywords:** septic embolism, natural valve, mitral valve, corynebacterium striatum, infectious endocarditis

## Abstract

*Corynebacterium striatum* is considered a rare pathogen in infective endocarditis (IE). *C. striatum* is a Gram-positive facultative anaerobic bacterium found in the environment and human flora. It is part of the microbiota of the skin and nasal mucosa of humans and has been increasingly reported as the etiologic agent of community-acquired and nosocomial diseases. A 91-year-old female patient was admitted to our clinic with complaints of increased fatigue for a week. Transthoracic echocardiography revealed a labile, echogenic appearance on the mitral valve that may be consistent with infective endocarditis, causing mitral regurgitation. Transesophageal echocardiography (TEE) confirmed this finding on the same day. In three-dimensional (3D) TEE, there was an oval mass of 1.9 cm × 1.1 cm at the level of the P2 scallop of the posterior mitral leaflet, and 1.0 cm of mobile vegetation was observed on it. Three serial blood cultures from peripheral vessels identified *C. striatum*. Antibiotic treatment of the patient was started with daptomycin 1 × 750 mg and meropenem 3 × 1 g. The cardiology team advised the patient to undergo early surgery, but the patient declined, and the case was followed up medically. On the 10th follow-up day, the patient had a speech disorder. Cerebral computed tomographic angiography showed an appearance compatible with a septic embolism in the left main cerebral artery. The patient's condition worsened throughout follow-ups, and she died on day 12. The purpose of presenting this case is to emphasize the importance of Corynebacterium species, which is a cause of rare native valve infectious endocarditis, and to show the difficulties in its treatment.

## Introduction

Infective endocarditis (IE) is a rare, life-threatening disease that takes a long time to treat. About 80% of instances of IE are caused by Staphylococci and Streptococci combined, whereas Enterococci are the third most common cause and are increasingly linked to healthcare contact [1]. *Corynebacterium striatum* is a Gram-positive facultative anaerobic bacterium found in the environment and human flora [2]. *C. striatum* is part of the microbiota of the skin and nasal mucosa of humans and has been increasingly reported as the etiologic agent of community-acquired and nosocomial diseases. It is considered a rare pathogen in IE. However, it is also possible that positive blood cultures of this pathogen are considered contamination, and therefore IE has been overlooked [3]. *C. striatum* infections have been reported in the literature, with bacteremia, central line infections, and occasionally endocarditis accounting for the majority of cases. The enhanced ease and precision of identifying Corynebacterium spp., including *C. striatum*, from clinical cultures is probably a contributing cause to the rise in the frequency of *C. striatum* infections that have been observed in recent years. We present a rare case of *C. striatum* seen in the natural mitral valve and with an aggressive course.

## Case presentation

**Figure 1:**
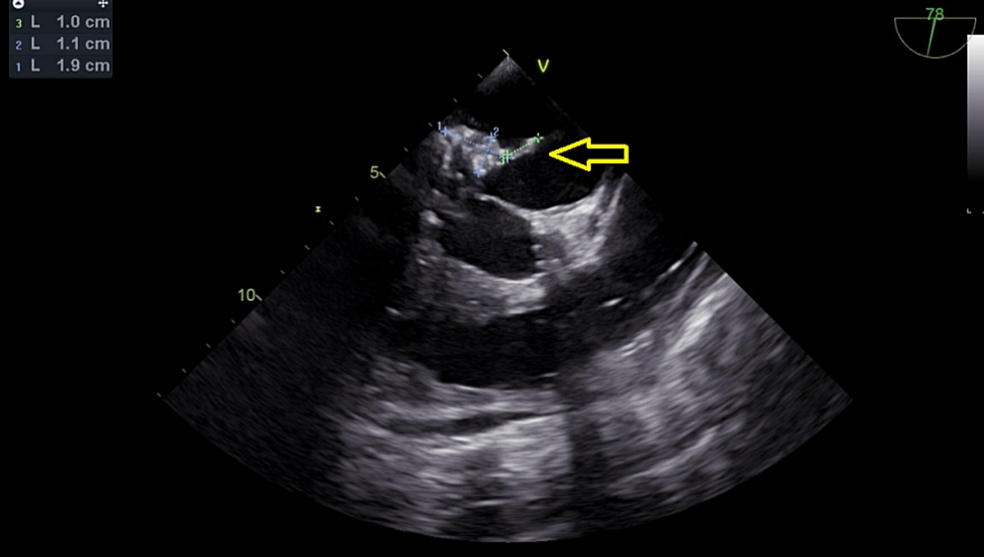
There was an oval mass of 1.9 cm × 1.1 cm at the level of the P2 scallop of the posterior mitral leaflet, and 1.0 cm of mobile vegetation was observed on TEE. Arrow shows vegetation on mitral valve.

A 91-year-old female patient was admitted to our clinic with complaints of increased fatigue for a week. A 91-year-old female patient was admitted to our outpatient clinic at Abdulhamid Han Research Hospital with a complaint of increased fatigue for the past week. The patient has no additional complaints other than mild shortness of breath on exertion. At her first examination, her blood pressure was 132/65 mmHg, her heart rate was 77 beats/min, and her body temperature was 36.8 °C. She had hypertension and chronic renal failure in her medical history. The patient had no history of recent catheter intervention or transfusion. Transthoracic echocardiography revealed a labile, echogenic appearance on the mitral valve that may be consistent with infective endocarditis, causing mitral regurgitation. Transesophageal echocardiography (TEE) confirmed this finding on the same day. In three-dimensional (3D) TEE, there was an oval mass of 1.9 cm × 1.1 cm at the level of the P2 scallop of the posterior mitral leaflet, and 1.0 cm of mobile vegetation was observed on it (Figure 1 and Video 1). In laboratory tests, troponin: 33 ng/L (threshold: 0-14), creatinine: 1.68 mg/dL, white blood cells (WBC): 8.92 103/mm^3^, and C-reactive protein (CRP): 11 mg/L (threshold: <5) were the findings. Blood cultures were taken from the peripheral upper extremity vein at half-hour intervals. *C. striatum* was isolated from serial blood cultures. Antibiotic treatment of the patient was started with daptomycin 1 × 750 mg and meropenem 3 × 1 g. The clinical situation was explained to the patient, and early surgery was recommended, but the patient refused surgical treatment, and the cardiology team's decision was to follow up medically. On the 10th day of follow-up, a speech disorder was observed in the patient. Cerebral computed tomographic angiography showed an appearance compatible with a septic embolism in the left main cerebral artery. The general condition of the patient deteriorated, her hemodynamics were unstable, and her Glasgow decreased in the follow-ups, and she died on the 12th day.

**Video 1:** TEE demonstration of mitral valve endocarditis due to Corynebacterium striatum

## Discussion

Commonly, Corynebacterium species isolated from blood cultures are disregarded as contaminants. Additionally, they are recognized as a rare pathogen in infectious endocarditis. When 30 cases of infective endocarditis due to Corynebacterium species were examined, 70% of the patients were elderly and associated with a prosthetic heart valve. 50% of them underwent surgical intervention, and the in-hospital mortality rate was 13% [4]. In the five cases described in the literature of cardiac device-related infective endocarditis caused by this agent, early removal of the pacemaker was performed with good results. One of the cases was removed after reinfection under medical treatment [5]. Most of the strains of *C. striatum* characterized were resistant to antimicrobials commonly used to treat Gram-positive organisms, such as penicillin, ceftriaxone, meropenem, clindamycin, and tetracycline. Although *C. striatum* exhibits limited sensitivity compared to other Corynebacterium species, it is frequently sensitive to vancomycin, linezolid, and daptomycin [6]. *C. striatum* is a pathogen that is gaining recognition because of its ability to resist many drugs, and it has been found to be linked with various forms of infection. Our case is an example of an aggressive course in the natural mitral valve. The purpose of presenting this case is to emphasize the importance of Corynebacterium species, which is a cause of rare native valve infectious endocarditis, and to show the difficulties in its treatment.

## Conclusions

Corynebacterium striatum is a pathogen to be considered in both native valve and prosthetic valve endocarditis and device infections. It should be noted that this bacterium may be sensitive to vancomycin, linezolid, and daptomycin, and prompt and effective treatment is important in case management. The surgical decision should be made in a timely manner in cases of valve failure that causes hemodynamic effects, large vegetations that may cause septic embolism, and cases resistant to medical treatment.

## References

[1] Holland TL, Baddour LM, Bayer AS, Hoen B, Miro JM, Fowler VG Jr (2016). Infective endocarditis. Nat Rev Dis Primers.

[2] Bernard K (2012). The genus corynebacterium and other medically relevant coryneform-like bacteria. J Clin Microbiol.

[3] Rasmussen M, Mohlin AW, Nilson B (2020). From contamination to infective endocarditis-a population-based retrospective study of Corynebacterium isolated from blood cultures. Eur J Clin Microbiol Infect Dis.

[4] Bläckberg A, Falk L, Oldberg K, Olaison L, Rasmussen M (2021). Infective endocarditis due to Corynebacterium species: clinical features and antibiotic resistance. Open Forum Infect Dis.

[5] Serpa Pinto L, Dias Frias A, Franca M (2021). Corynebacterium striatum cardiac device-related infective endocarditis: the first case report in a patient with a cardiac resynchronization therapy Defibrillator device and review of the literature. J Med Cases.

[6] McMullen AR, Anderson N, Wallace MA, Shupe A, Burnham CA (2017). When good bugs go bad: epidemiology and antimicrobial resistance profiles of Corynebacterium striatum, an emerging multidrug-resistant, opportunistic pathogen. Antimicrob Agents Chemother.

